# Survey of the Knowledge and Use of Antibiotics among Medical and Veterinary Health Professionals and Students in Portugal

**DOI:** 10.3390/ijerph18052753

**Published:** 2021-03-09

**Authors:** Ana Marta-Costa, Carla Miranda, Vanessa Silva, Adriana Silva, Ângela Martins, José Eduardo Pereira, Luis Maltez, Rosa Capita, Carlos Alonso-Calleja, Gilberto Igrejas, Patrícia Poeta

**Affiliations:** 1 Centre for Transdisciplinary Development Studies (CETRAD), University of Trás-os-Montes and Alto Douro (UTAD), 5000-801 Vila Real, Portugal; amarta@utad.pt; 2 Microbiology and Antibiotic Resistance Team (MicroART), Department of Veterinary Sciences, University of Trás-os-Montes and Aflto Douro, 5000-801 Vila Real, Portugal; carlisabelmi@utad.pt (C.M.); vanessasilva@utad.pt (V.S.); adriana.silva95@gmail.com (A.S.); jeduardo@utad.pt (J.E.P.); lmaltez@utad.pt (L.M.); 3 Associated Laboratory for Green Chemistry (LAQV-REQUIMTE), University NOVA of Lisboa, Caparica, 2829-516 Lisboa, Portugal; gigrejas@utad.pt; 4 Department of Genetics and Biotechnology, University of Trás-os-Montes and Alto Douro, 5000-801 Vila Real, Portugal; 5 Functional Genomics and Proteomics Unit, University of Trás-os-Montes and Alto Douro, 5000-801 Vila Real, Portugal; 6 Animal and Veterinary Research Center (CECAV), University of Trás-os-Montes and Alto Douro, 5000-801 Vila Real, Portugal; angela@utad.pt; 7 Department of Zootechnics, University of Trás-os-Montes and Alto Douro (UTAD), 5000-801 Vila Real, Portugal; 8 Department of Food Hygiene and Technology, Veterinary Faculty, University of León, E-24071 León, Spain; rosa.capita@unileon.es (R.C.); carlos.alonso.calleja@unileon.es (C.A.-C.); 9 Institute of Food Science and Technology, University of León, E-24071 León, Spain

**Keywords:** antibiotics, antimicrobial resistance, AMR, awareness, stewardship, One Health, questionnaire

## Abstract

Antimicrobial resistance (AMR) is an urgent and complex problem worldwide, exacerbated by the frequently inappropriate use of antibiotics. The purpose of this study was to survey the levels of knowledge and awareness about antibiotic use and stewardship, among human and veterinary health professionals or students in Portugal, and the associations between antibiotic knowledge factors and socio-professional groups. In cross-sectional survey design, a total of 449 online structured questionnaires were completed in 2018–2019. The statistical analysis was performed dividing the respondents into four groups, A (undergraduate students), B (PhD students and researchers), C (lecturers), and D (technicians and other occupation). Among all respondents, 17% (*n* = 75) revealed some gap in knowledge about antibiotic resistance and the antibiotics that should be administered for different infection types (bacterial, viral, or fungal). Of the 159 pet owners among the respondents, only half had administered antibiotics to their animal and 64% (*n* = 102) knew that veterinary prescription is mandatory when administering antibiotics to animals. All groups statistically agreed that the AMR is a major public health problem and the antibiotics should be administrated for bacterial infections and used until the whole pack has been finished (*p* = 0.00). As expected, only groups B and C demonstrated a higher level of knowledge to recognize the antibiotic name and their active ingredient than undergraduate students (*p* = 0.00). About the antibiotic use on pets, only group B was statistically significant to no used antibiotics on their pets (*p* = 0.00). However, groups A, C, and D were statistically significant for the knowledge about the mandatory veterinarian prescription and groups C and D were significantly statistics for fully aware of the transmission of bacteria between animals and humans. In conclusion, in matters related to AMR, the behavior, education, and training of the general public and health professionals, including those who prescribe antibiotics for humans and animals, need to be improved.

## 1. Introduction

The use of antibiotics has brought about a revolution in human and veterinary medicine and has contributed to saving millions of lives [1]. With the continued use of antibiotics, which includes overprescribing them, there is an increasing threat that the effectiveness of these drugs will be compromised because of the rise in bacterial resistance to antibiotics [1,2]. Any antibiotic use is indeed correlated strongly with the development of antimicrobial resistance (AMR). In only eight decades of antibiotic use, many bacterial infections which were previously easy to treat are becoming untreatable and some bacteria are now inherently resistant to antibiotics [3,4,5], corresponding to an increase of morbidity and mortality from secondary microbial infections [3]. The adequate management of infections and optimization and reduction of anti-infective therapy is necessary to combat worldwide microbial resistance. For example, in intensive care units, antimicrobial therapy is high [6,7]. In France, 41% of junior physicians acknowledged prescribing antibiotics more often than necessary [7]. On the other land, the use of antimicrobial agents in veterinary medicine is suspected of being an important factor in the development of antimicrobial-resistant organisms in humans, because of the close contact of companion animals with humans [8,9]. A recent study reported the scarce data about the number of used antibiotic in pets, as well as, its AMR problem, relative to food-producing animals. In addition, this study highlighted the increase of multidrug-resistant bacteria, such as enterococci and methicillin-resistant staphylococci in European pets [10].

In recent years, multiple international strategies and campaigns have been developed, and recommendations have been published to reduce or at least stabilize levels of AMR [2,11,12]. This happened after recognizing the multifaceted nature of the problem and that the solutions will require active efforts by practitioners of both human and veterinary medicine [13,14]. Jit et al. [15] also highlighted the importance of government interventions to reduce antibiotic resistance by incentivizing antibiotic development, prudent antibiotic use, infection control, and deployment of partial substitutes, such as rapid diagnostic tests and vaccines. Additionally, it is aimed at all public health authorities, practicing physicians, veterinarians, and other stakeholders involved in managing antimicrobial resistance, to ensure that all antimicrobials are used prudently both in human and veterinary medicine. These WHO recommendations were followed by several countries worldwide to develop their own antimicrobial programs [12].

A key public health priority on a global scale is thus to conserve antibiotics by optimizing how they are prescribed and taken in order to reduce AMR spread [4] and to introduce educational initiatives to encourage the coherent and proper use of antibiotics [16,17]. Future research may be needed to evaluate the prescription of antibiotics in both regular and emergency services, as well as on the knowledge and awareness of health professionals [17]. For example, the promotion of appropriate antibiotic stewardship for pet owners and vets may offer a viable pathway for planning interventions, benefitting from synergies with other interventions that target prescribers [9]. Most UK future health professionals, students on different courses like medicine, pharmacy, nursing, physician associate, dentistry, and veterinary medicine, want more education on antibiotic use and AMR for their future work [18].

Although the causes of AMR are complex, the main factors described as being responsible for AMR encompass inappropriate prescription practices, inadequate animal owners education, limited diagnostic facilities, unauthorized sale of antimicrobials, lack of appropriate functioning drug regulatory mechanisms, and non-human use of antimicrobials, such as in animals [3]. Several investigations in different countries have approached different population groups about some of these AMR-related issues, but all targeted one specific group. In a One Health approach, it is necessary to gather information about AMR knowledge and awareness from several target groups in the same population. The aim of this cross-sectional design study was to evaluate the level of knowledge and awareness of antibiotic use, resistance, and stewardship among current and future health professionals in human and veterinary medicine in Portugal. The data were analyzed to investigate statistic differences between the antibiotic knowledge factors and each of the studied socio-professional groups.

## 2. Materials and Methods

### 2.1. Selection of the Study Population

The study was carried out in northern Portugal. Two public universities were selected based on their size and location to ensure a diversity of socioeconomic contexts. One university offering both veterinary and medical courses is located in a metropolitan area near the coast that is very attractive to incomers, partly due to its better transport access and higher population density. The other university is set in a rural inland context, where population densities are lower, but has the strong attraction of the integrated veterinary medicine master’s degree course it offers. Combined, about 3550 students were enrolled in the veterinary and medical courses at the two institutions for the academic year of 2017/2018 [19].

### 2.2. Research Design

The conceptual framework that represents this work was based on the findings of previous studies in this area, as described by Dyar et al. [17], Napolitano et al. [20], Rábano-Blanco et al. [21], and Xia et al. [22], on the diagnosis for antibiotic use and management by the health community and how knowledge and concerns surrounding the issue need to be evaluated to try to raise awareness of antibiotic use, resistance, and stewardship. To identify the professional groups to include, the advantages of targeting individual researchers and institutions from academia, industry, and practitioners [14], like students, teachers, researchers, and technicians, were taken into account.

### 2.3. Sampling Techniques and Sample Determination

The data were obtained by means of a sample survey conducted in the two higher education institutions of northern Portugal. These institutions were also selected because they include future health professionals (students of the above courses), and working professionals (researchers, doctors, veterinarians, technicians) with varying levels of responsibility and experience in the subject, some of whom are also pet owners. The differences between perspectives and behaviors of both groups could hence allow for a comparative analysis. The study population includes professionals with different profiles, allowing for representativeness of the sample to be considered in terms of gender and age.

No register of the population under study was available to support the sampling, other than the students’ enrolment on the mentioned courses, so non-random sampling techniques were used [23]. In addition, the option of trying to contact as many individuals in the target population as possible was chosen as a strategy, as indicated by Maciel et al. [24]. This was done by sending an institutional e-mail to the addresses of the institutions and course directors, asking for the questionnaire to be forwarded to all students and collaborators affiliated with the institution and implicated in animal and human healthcare courses.

### 2.4. Data Collection

Data were collected through the realization of an online questionnaire entitled “Antibiotic use survey”. In the absence of a standardized enquiry tool, the questionnaire was developed and built by a multidisciplinary group of health students and experts on responsible antimicrobial use, taking into account the results from previous studies [25,26], as well as, the knowledge gaps in the use and management of antibiotics encountered in their own work.

The structured questionnaire consisted of 28 closed-ended questions (dichotomous or multiple choice) organized into two main sections (Tables S1 and S2). The first section included the respondent characterization, according to the Hill and Hill [23] recommendations, and questions to evaluate the general level of knowledge of antibiotics use by all the participants. The second section, intended only for those respondents with pets, addressed specific knowledge and awareness regarding the use of antibiotics in pets. The suggestions made by Thayer-Hart et al. [27] and Maciel et al. [24], regarding the survey of more specific aspects into the advanced stage of the questionnaire, were thus followed. The questionnaire was pretested with a group of researchers and students to check the quality of the questions, the proper functioning of the entire response system, and the collection of data from the server as suggested by Terrível [28].

All respondents, current and future health professionals of the study population from the respective university departments of human and veterinary health, were invited to participate in the study by its institutional email. The questionnaire was preceded by a brief introductory note, which set out the request for cooperation, the reason for the survey, a description of the questionnaire, the institution conducting the investigation, ensuring the anonymity. At the end of section I of the questionnaire, the respondents that had pets continued to section II. Whoever did not identify themselves as pet owners were directed politely to the end of the questionnaire. The survey was self-completed in 2018–2019 and the participation was voluntary without any compensation. All subjects gave their informed consent for inclusion before they participated in the study. The study was conducted in accordance with the Declaration of Helsinki, and the protocol was approved by the Ethics Committee of University of Trás-os-Montes e Alto Douro (EC-UTAD, number 29-CE-2020).

### 2.5. Statistical Analysis

Descriptive statistics, primarily, were used to summarize all information, presenting the results as number and percentage. In the second step of statistical analysis, a proportion of categories was performed in comparison with the socio-professional group responses by qui-square (X^2^) tests, using the JMP 7.0 (SAS, Cary, USA, 2007) software, in which the respondents were joined into four socio-professional groups, A (undergraduate students), B (PhD students and researchers), C (lecturers), and D (technicians and other occupation).

## 3. Results

A total of 449 individuals completed the online version of the “Antibiotic use survey” questionnaire. Group A was the most representative and included undergraduate students from all the academic years, followed by groups C and D at two universities in the north of Portugal that provide courses in human and veterinary health (Table 1). The PhD students and researchers in group B were the least representative group. Of all respondents, 69% were females and 28% males, and 38% were between 18 and 30 years old.

In all socio-professional groups, respondents of female gender were predominant, in which the statistical analysis (Table 2) showed that the socio-professional groups, such as A, C, and D (*p* = 0.00) were more likely to be female gender. The 18–30-year-old respondents were more likely to be found in the undergraduate group than in other analyzed health professional groups (*p* = 0.00). However, of the 449 individuals, the undergraduate students corresponded to a 5% response rate, and groups B, C, and D corresponded to 34%, 19%, and 62% response data, respectively (Table 3). Additionally, PhD students plus researchers (group B) and lecturers (group C) were significantly statistic to age (*p* = 0.01 and *p* = 0.00, respectively).

### 3.1. General Knowledge and Personal Use of Antibiotics

All results obtained on the general level of knowledge of antibiotics use and some of the care (or lack of care) that respondents took with antibiotics are shown in Table S1. Among all 449 survey respondents, 83% of them (374 respondents) considered antibiotic resistance to be a major public health problem. However, the remaining respondents (17%), including 11% of all the lecturer, 13% of all the PhD students plus researchers and technicians plus other occupation, and 24% of all the undergraduate students surveyed, either admitted to a lack of sufficient knowledge on the subject or thought that the issue is not relevant (Table 4). Seventy-seven respondents (17%) belonging to the various professional groups surveyed even indicated that antibiotics should be administered for any type of infection, whether bacterial, viral, or fungal, although 62% (*n* = 280) of respondents thought antibiotics should be administered for bacterial infections. Revealingly, 9% (*n* = 42) of the respondents considered the prescription of antibiotics by a doctor to be a sign of competence to treat a patient presenting with fever, headaches, runny nose, cough, and muscular pains for three days. Most respondents consider re-evaluation (*n* = 206, 46%) or test analysis (*n* = 192, 43%) as the appropriate course of action.

Three quarters of the respondents (*n* = 336, 75%), which included individuals from all groups in the population, stated that they had not bought and/or taken an antibiotic without it having been prescribed by a doctor, in opposite a quarter (*n* = 111, 25%), and 12% (*n* = 54) stated that they use unfinished boxes of antibiotics to treat relatives and/or friends, and this occurred several times in the year.

Most respondents, 88% (*n* = 395), indicated that the prescribed antibiotic should be used to the end of the pack or course prescribed, but 18% (*n* = 79) considered it appropriate to keep any leftover antibiotic for future use or to throw it away, while only 60% (*n* = 270) claimed to always check the pack for instructions on how to use the antibiotics. Knowing the socio-professional groups represented in the study has responsibilities and education in the field. Moreover, there may be several reasons for these data, and a closer look allowed the following factors to be distinguished: (i) Medical instructions for taking the antibiotic correctly were always followed by 53% (*n* = 238) of respondents but this proportion rises to 92% (*n* = 409) if the statement is qualified by the word “sometimes”; (ii) Instructions on the leaflet in the antibiotic pack were fully heeded by only 46% (*n* = 207) of respondents; (iii) Experience and/or confidence with the use of different antibiotics varies as 64% (*n* = 286) of respondents have never had to take more than one antibiotic for treatment of a disease; and (iv) A large proportion of respondents (*n* = 286, 64%) recognize the names of some antibiotics and their active ingredient, which may indicate the level of knowledge and possibly autonomy when taking the product.

Respondents usually bought their antibiotics from community pharmacies (*n* = 247, 55%) or through their doctor (*n* = 185, 41%). Only 1% (*n* = 10) of the respondents obtained their antibiotics by other sources. Regarding the antibiotics taken by respondents in the last year, responses were split almost equally between those who took antibiotics between one to five times (*n* = 222, 49%) and those who had not taken any product of this type (*n* = 215, 48%).

The statistical analysis of general knowledge and personal use of antibiotics relative to analyzed socio-professional groups was presented in Table 4. All groups statistically agreed that the bacterial resistance to antibiotics is a major public health problem (*p* = 0.00) and the antibiotics should be administrated for bacterial infections (*p* = 0.00) and used until the whole pack has been finished (*p* = 0.00). Additionally, all groups (*p* = 0.00) agreed that the elimination of leftover antibiotics should be returned to the pharmacy or never left unused. Although the four socio-professional groups showed a significant statistic when the respondent or a family member have been ill for 3 days, 60% of lecturers agree to return to the consultation after 2–3 days to be reassessed while 55% of undergraduate students wait for the doctor to test before prescribing an antibiotic.

A majority of respondents of each group do not get antibiotics without a prescription and these usually get them in community pharmacies or through their doctor, showing statistically significant results. As expected, only groups B (doctoral students plus researchers) and C (lecturers) were statistically significant to recognize the antibiotic name and their active ingredient. In spite of all groups being significant statistic (*p* = 0.00), groups B and C were more likely to follow the totally clear instructions for antibiotic use than groups A and D. However, 35% of lecturers (*p* = 0.00) and 32% of undergraduate students (*p* = 0.00) had correctly taken a pack of antibiotics prescribed by the doctor but needed a different antibiotic to continue treatment.

### 3.2. Specific Knowledge and Experience of the Use of Antibiotics to Treat Pets

One hundred and fifty-nine (35%) respondents had pets and their answers to questions regarding antibiotics use in pets are summarized in Table S2. In addition, undergraduate students (*p* = 0.00) and technicians plus health professionals with other occupations (*p* = 0.00) were less likely to have pets than other groups (Table 4). About half of the pet owners (*n* = 74, 47%) had already used antibiotics for their animals. The proportions of specific knowledge and experience of the use of antibiotics to treat pets by the socio-professional group responses (Table 5) showed that only group B was statistically significant to not use antibiotics on their pets. When asked whether non-prescribed antibiotics had been given to pets, 90% (*n* = 143) answered no and only 8% (*n* = 13) of the pet owners answered in the affirmative (Table S2).

Only 60% (*n* = 96) of respondents with pets completed the course of treatment recommended by the veterinarian and about 81% (*n* = 129) gave the recommended doses (Table S2). However, it should be noted that one quarter of the pet owners did not answer these compliance-related questions (29% and 18% for the duration and doses of treatment, respectively). Regarding the regularity in prescribing antibiotics for pets in the last year, 44% (*n* = 70) of pet owners indicated that the situation occurred only once, 9% (*n* = 15) a frequency of up to five times. The statistical analysis confirmed that all socio-professional groups, only stopping the pet’s antibiotic treatment when indicated by the veterinary doctor, showing a statistically significant results (Table 5). Although some categories have a reduced number of answers, the undergraduate students with pets were significantly statistic (*p* = 0.00) to not change the recommended dosage or antibiotic during the treatment.

Among the pet owners, only 64% (*n* = 102) thought that the use of antibiotics in animals is only allowed with prescription from a veterinarian. However, only three socio-professional groups (A, C, and D) were significantly statistic with the knowledge about the veterinarian prescription. In addition, 40% (*n* = 63) did not always disclose to the veterinarian their availability/willingness to correctly administer the antibiotic to their pet, including some (12%, *n* = 19) who indicated that they did not think this factor was important for the success of the treatment. The unavailability of information (e.g., posters or free flyers) on the proper use of antibiotics in veterinary clinics attended was reported by 46% (*n* = 73) of respondents. Only 41% (*n* = 66) of the respondents who owned pets had a notion of the possibility of spreading antibiotic resistant bacteria from animals to humans and vice versa, which was surprising considering the different socio-professional groups represented in the survey. Of these, lecturers and technicians plus professional with other occupations of groups C and D (74%, *p* = 0.00 and 33%, *p* = 0.03, respectively) were significantly statistic for being fully aware of the transmission of bacteria between animals and humans.

## 4. Discussion

Through a consultation with stakeholders in human and veterinary health by institutional email, we sought to survey the knowledge and awareness of antibiotic use, resistance, and stewardship among current and future health professionals. The findings of the survey showed most of the respondents in different stakeholder groups have correct behavior and perceptions about antibiotics use and its importance. However, the answers revealed gaps in this kind of knowledge and inappropriate behavior when taking and/or prescribing antibiotics in a small proportion of the sample. While these current and potential professionals are generally familiar with antibiotics, awareness of antibiotic resistance was insufficient in more than a quarter of the sample.

Antibiotic resistance is an emerging concern with serious public health repercussions in terms of morbidity and mortality [21] and is regarded as a major worldwide health crisis [9,29,30]. This perspective was shared by 83% of respondents to the survey conducted as part of this study. The remaining respondents, which included 23% of surveyed students, either revealed a lack of sufficient knowledge on the subject or they thought that the problem is not relevant. These findings are in line with those obtained by Rábano-Blanco et al. [21] for a similar group (students), which points to the need for more education on antibiotics and infection control in degree courses, especially as students are future clinicians, teachers, and researchers who will be at the forefront in educating the general public. In addition to the minor importance some respondents attached to the problem of bacterial resistance to antibiotics, the incorrect use of antibiotics, such as keeping remaining stocks for future use or throwing them away, was perceptible in this study. This situation appears more serious in light of the socio-professional situations of the respondents. Rábano-Blanco et al. [21] also highlighted the knowledge and attitudes of healthcare professionals, pointing out that not only does poor knowledge or a negative attitude towards antibiotics lead to bad clinical practices, but also practical skills are not always a reflection of knowledge. This situation was called the “theory-practice gap”. Additionally, Scaioli et al. [26] recommended creating more awareness on this topic during degree courses to prepare for when students become medical doctors and are able to prescribe these drugs. One effective strategy recommended by Alzahrani et al. [17] and O’Neill [29] to combat incorrect antibiotics use consists in continuing education to improve knowledge and awareness of both professionals and the general population.

Wayne et al. [25] stated that there is overuse and inappropriate use of antibiotics in veterinary medicine, and suggested that veterinarians should engage in discussions of clinically applicable guidelines for appropriate antibiotic use. In situations of our study where incorrect practices were indicated, it was noted that they occurred in similar proportions in the different professional subgroups surveyed, suggesting that the lack of knowledge and attention shown by the groups with the greatest responsibilities (e.g., lecturers, researchers, and clinicians) might be reflected throughout the knowledge transfer chain. These findings are very useful considering that most antibiotic resistance control strategies recommend education of the general population, mainly by healthcare workers. Rábano-Blanco et al. [21] also reported that nursing students assumed great difficulties in the selection of the best antibiotic for a specific infection. Most French junior physicians (93%) of family medicine and several medical specialties answered to being aware of the risk of bacterial resistance on the web questionnaire but in their workplace, only 74% recognized it a major problem [7]. In our study, statistically significant differences were found (Table 4), the most of undergraduate students (Group A) agreed that antibiotics are useful for bacterial infection and not for viral or fungal infections (50%, *n* = 82, *p* = 0.00), and the bacterial resistance to antibiotics is a major public health problem (76%, *n* = 124, *p* = 0.00). Another study including university students demonstrated that 63% kept antibiotic in their home and 28% were leftover from a previous prescription by a doctor but 69% had purchased antibiotics over the counter, consequently, students keeping antibiotics at home were more likely to engage in self-medication when ill and as a prophylactic measure than other students [31]. However, students of a school of medicine or those who took an antibiotic course recently had a lower probability of taking antibiotics only under prescription [26]. In opposite, only 15% (*n* = 24, *p* = 0.00) of undergraduate students and 10% (*n* = 5, *p* = 0.00) of doctoral students plus researchers (group B) of our analyzed health universities stored leftover antibiotics for future use. In addition, a majority of respondents of these two groups demonstrated not getting antibiotics without prescription and these usually were obtained in community pharmacies or through your doctor.

The realization of testing of antimicrobial susceptibility to identify possible antibiotic resistances before the antibiotic administration is essential, as well as the participation and collaboration of health professionals in integrated programs related to antibiotic resistance or other epidemic-vigilance studies [32]. Igrejas et al. [14] highlighted that active participation in the National Action Plan for Antibiotics Use Reduction in Animals would help to trace, validate, and requisition veterinary prescription, harmonizing the register of all medicines administered at farms and pet clinics. With adequate support and training of all professions dealing with animal health and production, better antibiotics selection and use will be encouraged, and innovations and alternatives can be explored. Our study confirmed this need; only the lecturers reported a constant discussion with the veterinarian on their availability/willingness to correctly administer the antibiotic to their pet. Moreover, the unavailability of information (e.g., posters or free flyers) on the proper use of antibiotics in veterinary clinics attended was negatively reported by undergraduate students (*p* = 0.00). Optimizing the prescription of antibiotics to reduce the spread of AMR is a key public health priority both globally and nationally and all clinicians surveyed considered that prescribing an antibiotic to a patient may influence the onset of resistance [4]. Consistent with this, none of the respondents leveraged antibiotic prescription as a way to gain the trust of pet owners. This is despite the fact that pet owners have been found to have substantial influence over veterinary decision making on antibiotic use [9]. Even in the small group surveyed, some of the clinicians reported that they try to adapt the antibiotic they prescribe to the situation and usually opted for a broad-spectrum antibiotic, or prescribed antibiotics as a way of preventing infections or for non-therapeutic purposes. According to the data reported by Borek et al. [4], in England, 81% of antibiotics were prescribed in primary care in 2017 and up to 23% of these were estimated to be inappropriately prescribed. Therefore, there is a need to focus efforts on prudent antibiotics use to prevent the emergence of resistant strains, and this problem is the responsibility of health professionals, from clinicians, health technicians, and even students in training, as well as the general population, as all are implicated directly or indirectly in inappropriate antibiotic use.

This type of survey has many advantages, but mainly that it is economical and efficient in terms of the time needed for sending out questionnaires and collecting data [28]. The limitations associated with the problems of population coverage encountered by Couper [33] were readily overcome by limiting the survey to populations where internet access was guaranteed. However, online questionnaires have limitations. Several studies point to a lower response rate in online compared to traditional survey modalities [33,34], but others have demonstrated that fewer questions remain unanswered [35]. Terrível [28] reported problems with data reliability and origin. Measures to overcome these issues were to emphasize the formal character of the survey through the presentation of the study objectives and the institutions involved; by the development of a clear, well-timed, attractive, and easy-to-fill-in survey model in order to obtain the highest possible response rate.

## 5. Conclusions

The true scope of antimicrobial stewardship surveys among health professionals is uncertain, but it is a reasonable starting point as a way to tackle the problem from both human and pet welfare standpoints. In the present study, many factors related to antibiotic prescription have been identified as abnormal situations or even misuse of these substances, like stopping the antibiotic before finishing the box or course prescribed, reusing leftover substances in later situations and the lack of explanation of the importance of the correct dose by the doctor.

The control and management of antibiotic prescriptions should also be coupled with top-down strategies to enable and enhance monitoring of prescribing data at a national level and to improve engagement of the healthcare stakeholders (clinicians, pharmacists, researchers, teachers, students, among others) with antimicrobial stewardship training and resources. The competent authorities have practical experience and knowledge of national antimicrobial stewardship interventions and policy, and can refine and adapt the approaches. However, both these mechanisms are slow and long-term processes, the results of which are not immediate in terms of the participatory involvement of stakeholders and the operational effects of an active public participation policy. This can still be a viable way towards more effective antimicrobial stewardship as it may help reduce conflicts within the healthcare community, particularly in an interdisciplinary context.

In conclusion, this study identified a number of shortcomings in prescribing and taking antibiotics. Although groups B and C demonstrated a higher level of knowledge about antibiotic use, antimicrobial resistance concern, and recognizing the antibiotic name and their active ingredient than undergraduate students (group A), the respondents demonstrated to lack information and formation. More representative studies are urgently needed to understand the real-national and real-world situation about the prescription and use of antibiotics by medical and non-medical populations, respectively. The solution to this problem requires a concerted One Health approach to mitigate future risks to humans, animals, and the environment.

## Figures and Tables

**Table 1:** Profile of the population of survey respondents by age, gender, and socio-professional group.

Socio-Professional Group	Age									Total
	18–30 Years		31–40 Years		41–50 Years		≥51 Years		without Answer	Nr. (%)
	F	M	F	M	F	M	F	M		
**A**	110	26	12	8	3	1	2	0	2	164 (36)
**B**	7	5	12	9	8	5	3	2	2	53 (12)
**C**	3	1	18	9	38	18	19	15	5	126 (28)
**D**	15	4	23	6	24	10	17	4	3	106 (24)
**Total (%)**	135 (30)	36 (8)	65 (14)	32 (7)	73 (16)	34 (8)	41 (9)	21 (5)	12 (3)	449(100)

F, female; M, male. A, undergraduate students; B, PhD students and researchers; C, lecturers; D, technicians and other occupations.

**Table 2:** Proportions of gender and age by the socio-professional group responses.

Variable	Total Number	Category	Socio-Professional Group			
			A	B	C	D
			Nr. (%)	Nr. (%)	Nr. (%)	Nr. (%)
Gender	438	*p* value	***0.00***	***0.21***	***0.00***	***0.00***
		Female	127 (78)	30 (59)	79 (65)	79 (77)
		Male	35 (22)	21 (41)	43 (35)	24 (23)
Age	449		***0.00***	***0.01***	***0.00***	***0.12***
		18–30 years	138 (84)	12 (23)	5 (4)	19 (18)
		31–40 years	20 (12)	23 (43)	29 (23)	31 (29)
		41–50 years	4 (3)	13 (25)	57 (45)	34 (32)
		≥51 years	2 (1)	5 (9)	35 (28)	22 (21)

A, undergraduate students; B, PhD students and researchers; C, lecturers; D, technicians and other occupations.

**Table 3:** Response rate obtained in this study.

Socio-Professional Group	Contacts Number	Respondents Number	Response Rate (%)
A (Undergraduate students)	3550	164	5
B (PhD students and researchers)	156	53	34
C (Lecturers)	665	126	19
D (Technicians and Other occupations)	170	106	62

**Table 4:** Proportions of general knowledge and personal use of antibiotics by the socio-professional group responses.

Variable	Total Number	Category	Socio-Professional Group			
			A	B	C	D
			Nr. (%)	Nr. (%)	Nr. (%)	Nr. (%)
**What is your view of bacterial resistance to antibiotics?**	447	*p* value	***0.00***	***0.00***	***0.00***	***0.00***
		Major public health problem	124 (76)	46 (87)	112 (89)	92 (87)
		Do not know enough to answer	32 (20)	6 (11)	10 (8)	13 (12)
		Maybe worrying but it does not matter greatly	6 (4)	1 (2)	4 (3)	1 (1)
**Antibiotic should be administered for:**	442		***0.00***	***0.00***	***0.00***	***0.00***
		Bacterial infections	82 (50)	39 (73)	95 (77)	64 (63)
		Bacterial and fungal infections	12 (7)	2 (4)	7 (6)	9 (9)
		Bacterial and viral infections	15 (9)	2 (4)	3 (2)	5 (5)
		Viral infections	15 (9)	2 (4)	7 (6)	6 (6)
		All	39 (25)	8 (15)	12 (9)	18 (17)
**Prescribed antibiotic should be used:**	438		***0.00***	***0.00***	***0.00***	***0.00***
		Until the whole pack/course has been finished	135 (83)	47 (90)	115 (95)	98 (95)
		Until you feel better	13 (8)	3 (6)	2 (2)	2 (2)
		You are not sure	14 (9)	2 (4)	4 (3)	3 (3)
**Any leftover antibiotic should be:**	443		***0.00***	***0.00***	***0.00***	***0.00***
		Thrown in the garbage	10 (6)	5 (10)	7 (6)	10 (10)
		Returned to the pharmacy	69 (42)	29 (55)	54 (43)	47 (45)
		Stored for future use	24 (15)	5 (10)	12 (10)	6 (6)
		Never left unused	60 (37)	13 (25)	51 (41)	41 (39)
**For 3 days you or a family member have had a fever, headache, runny nose, cough and muscle pain. When you see a doctor, do you…?**	440		***0.00***	***0.00***	***0.00***	***0.00***
		Agree to return to the consultation after 2–3 days to be reassessed	58 (36)	25 (47)	74 (60)	49 (48)
		Consider the doctor competent if s/he prescribes an antibiotic immediately	15 (9)	5 (9)	11 (9)	11 (10)
		Wait for the doctor to test you before prescribing an antibiotic	88 (55)	23 (44)	38 (31)	43 (42)
**Have you ever obtained/taken an antibiotic without it being prescribed by a doctor?**	447		***0.00***	***0.00***	***0.00***	***0.00***
		No	126 (77)	38 (72)	91 (72)	81 (77)
		Yes	37 (23)	15 (28)	35 (28)	24 (23)
**If you answered Yes to Q9, how many times has this happened in the last year?**	111		***0.02***	***0.05***	***0.00***	***0.00***
		Once	25 (69)	10 (77)	29 (94)	19 (83)
		More than 2 times	11 (31)	3 (23)	2 (6)	4 (17)
**Where do you usually get antibiotics?**	442		***0.00***	***0.00***	***0.00***	***0.00***
		Through your doctor	74 (46)	18 (36)	42 (34)	51 (49)
		Community pharmacies	85 (52)	29 (58)	80 (64)	53 (50)
		Other	3 (2)	3 (6)	3 (2)	1 (1)
**When you are prescribed an antibiotic, does your doctor explain the importance of taking it correctly?**	442		***0.00***	***0.00***	***0.00***	***0.00***
		Sometimes	61 (38)	24 (45)	49 (39)	40 (39)
		Never	14 (9)	2 (4)	9 (7)	5 (5)
		Always	86 (53)	27 (51)	67 (54)	58 (56)
**Can you recognize the name of some antibiotics and their active ingredient?**	441		***0.10***	***0.00***	***0.00***	***0.06***
		No	71 (44)	13 (25)	29 (24)	42 (41)
		Yes	92 (56)	40 (75)	93 (76)	61 (59)
**Have you ever used leftover antibiotics to treat other family members and/or friends?**	442		***0.00***	***0.00***	***0.00***	***0.00***
		1–5 times	15 (9)	4 (7)	11 (9)	10 (10)
		Several times	7 (4)	1 (2)	5 (4)	1 (1)
		Never	140 (87)	48 (91)	108 (87)	92 (89)
**Do you check the pack for instructions on how to use the antibiotic?**	441		***0.00***	***0.00***	***0.00***	***0.00***
		Never	5 (3)	2 (4)	6 (5)	7 (7)
		Sometimes	64 (40)	14 (27)	44 (35)	29 (28)
		Always	91 (57)	36 (69)	75 (60)	68 (65)
**How clear are instructions for antibiotics use?**	439		***0.00***	***0.00***	***0.00***	***0.00***
		Not clear	1 (1)	2 (4)	1 (1)	2 (2)
		Partially clear	96 (60)	22 (41)	52 (43)	56 (54)
		Totally clear	63 (39)	29 (55)	69 (56)	46 (44)
**How many times in the last year have you taken antibiotics?**	442		***0.00***	***0.00***	***0.00***	***0.38***
		1–5 times	80 (49)	24 (45)	61 (50)	57 (54)
		More than 5 times	3 (2)	1 (2)	1 (1)	-
		None	80 (49)	28 (53)	59 (49)	48 (46)
**Have you ever correctly taken a pack of antibiotics prescribed by the doctor and had to continue treatment with a different antibiotic?**	446		***0.00***	***0.07***	***0.00***	***0.08***
		1–5 times	52 (32)	20 (38)	43 (35)	44 (42)
		None	112 (68)	32 (62)	82 (65)	62 (58)
**Do you have pets?**	449		***0.00***	***0.89***	***0.05***	***0.00***
		No	113 (69)	26 (49)	74 (59)	77 (73)
		Yes	51 (31)	27 (51)	52 (41)	29 (27)

A, undergraduate students; B, PhD students and researchers; C, lecturers; D, technicians and other occupations.

**Table 5:** Proportions of specific knowledge and experience of the use of antibiotics to treat pets by the socio-professional group responses.

Variable	Total Number	Category	Socio-Professional Group			
			A	B	C	D
			Nr. (%)	Nr. (%)	Nr. (%)	Nr. (%)
**Have you ever used antibiotics on your pet?**	**157**	***p* value**	***0.88***	***0.00***	***0.58***	***0.85***
		**No**	25 (49)	21 (78)	24 (46)	13 (48)
		**Yes**	26 (51)	6 (22)	28 (54)	14 (52)
**Have you ever medicated your pet with non-prescribed antibiotics?**	**156**		***0.00***	***0.00***	***0.00***	***0.00***
		**No**	48 (94)	24 (89)	49 (94)	22 (85)
		**Yes**	3 (6)	3 (11)	3 (6)	4 (15)
**Do you ever stop your pet’s antibiotic treatment?**	**113**		***0.00***	***0.00***	***0.00***	***0.00***
		**When indicated by the veterinary doctor**	31 (76)	11 (79)	40 (96)	14 (88)
		**Yes**	2 (5)	1 (7)	1 (2)	2 (12)
		**Yes, when my pet seems to be perfectly back to normal**	7 (17)	2 (14)	1 (2)	-
		**Yes, as soon as I notice improvements**	1 (2)	-	-	-
**Do you change the dosage or antibiotic during treatment?**	**131**		***0.00***	***(a)***	***(a)***	***(a)***
		**No**	45 (96)	19 (100)	46 (100)	19 (100)
		**Yes**	2 (4)	-	-	-
**How regularly over the past year has your veterinarian prescribed antibiotics for your pet?**	**85**		***0.01***	***(a)***	***0.00***	***0.09***
		**Once**	24 (73)	10 (100)	29 (88)	7 (78)
		**1 to 5 times**	9 (27)	-	4 (12)	2 (22)
**Do you know that the use of antibiotics in animals is allowed only with a prescription from a veterinarian?**	**145**		***0.01***	***0.10***	***0.00***	***0.02***
		**No**	15 (31)	8 (33)	15 (29)	5 (25)
		**Yes**	34 (69)	16 (67)	37 (71)	15 (75)
**Do you know that antibiotic-resistant bacteria can spread from animals to humans and vice versa?**	**147**		***0.11***	***0.23***	***0.00***	***0.03***
		**Yes. I’m fully aware of this fact**	10 (20)	12 (48)	37 (74)	7 (33)
		**I suspected that there might be some connection**	21 (41)	8 (32)	12 (24)	2 (10)
		**I had no idea**	20 (39)	5 (20)	1 (2)	12 (57)
**Do you usually discuss with your veterinarian your willingness and availability to administer the antibiotic to your pet correctly and on time?**	**120**		***0.34***	***0.90***	***0.00***	***0.21***
		**I do not consider this important for the success of the treatment**	11 (24)	3 (30)	3 (6)	2 (12)
		**Sometimes**	16 (35)	3 (30)	18 (38)	7 (44)
		**Yes. Always.**	19 (41)	4 (40)	27 (56)	7 (44)
**Does your usual veterinary clinic display information (e.g., posters, flyers) directed to pet owners about the proper use of antibiotics?**	**136**		***0.00***	***0.09***	***1.00***	***0.13***
		**No**	35 (76)	5 (29)	26 (50)	7 (33)
		**Yes**	11 (24)	12 (71)	26 (50)	14 (67)

A, undergraduate students; B, PhD students and researchers; C, lecturers; D, technicians and other occupations. (*a*), only one type of answers obtained.

## Data Availability

The data presented in this study are available on request from the corresponding author.

## Supplementary Materials

The following are available online at https://www.mdpi.com/1660-4601/18/5/2753/s1, Table S1: Questions performed in the first section of the questionnaire, including the respondent characterization and the general knowledge and personal use of antibiotics, for all the respondents, Table S2: Questions performed in the second section of the questionnaire, including specific knowledge and experience of the use of antibiotics to treat pets, for the pet owners.
