# Supplementary material for: Survey of the Knowledge and Use of Antibiotics among Medical and Veterinary Health Professionals and Students in Portugal
Source: Int J Environ Res Public Health. 2021 Mar 9;18(5):2753. doi: 10.3390/ijerph18052753 (PMC7967476; doi:10.3390/ijerph18052753)
Supplement: Supplementary file 1 [file ijerph-18-02753-s001.pdf]

International Journal of
Environmental Research and Public Health

MDPI

Supplementary Materials

Survey of the Knowledge and Use of Antibiotics among Medical and Veterinary Health Professionals and Students in Portugal

Ana Marta-Costa [1,†], Carla Miranda [2-4,†], Vanessa Silva [2,4-6], Adriana Silva [2,4-6], Ângela Martins [7,8], José Eduardo Pereira [2,3,7], Luis Maltez [2,3,7], Rosa Capita [9,10], Carlos Alonso-Calleja [9,10], Gilberto Igrejas [4-6] and Patrícia Poeta [2-4,*]

[1] Centre for Transdisciplinary Development Studies (CETRAD), University of Trás-os-Montes and Alto Douro (UTAD), 5000-801 Vila Real, Portugal; amarta@utad.pt (A. M-C.)
[2] Microbiology and Antibiotic Resistance Team (MicroART), Department of Veterinary Sciences, University of Trás-os-Montes and Alto Douro, 5000-801 Vila Real, Portugal
[3] Department of Veterinary Sciences, University of Trás-os-Montes and Alto Douro, 5000-801 Vila Real, Portugal carlisabelmi@utad.pt (C. M.); jeduardo@utad.pt (J. E. P.); lmaltez@utad.pt (L. M.); ppoeta@utad.pt (P. P.)
[4] Associated Laboratory for Green Chemistry (LAQV-REQUIMTE), University NOVA of Lisboa, Caparica, 2829-516 Lisboa, Portugal
[5] Department of Genetics and Biotechnology, University of Trás-os-Montes and Alto Douro, 5000-801 Vila Real, Portugal; vanessasilva@utad.pt (V. S.); adriana.silva95@gmail.com (A. S.); gigrejas@utad.pt (G. I.).
[6] Functional Genomics and Proteomics Unit, University of Trás-os-Montes and Alto Douro, Vila Real, Portugal
[7] Animal and Veterinary Research Center (CECAV), University of Trás-os-Montes and Alto Douro, 5000-801 Vila Real, Portugal
[8] Department of Zootechnics, University of Trás-os-Montes and Alto Douro (UTAD), 5000-801 Vila Real, Portugal; angela@utad.pt (A.M.)
[9] Department of Food Hygiene and Technology, Veterinary Faculty, University of León, E-24071 León, Spain; rosa.capita@unileon.es (R. C.); carlos.alonso.calleja@unileon.es (C. A.-C.).
[10] Institute of Food Science and Technology, University of León, E-24071 León, Spain
* Correspondence: ppoeta@utad.pt (P.P.)
† These authors have contributed equally to this work

International Journal of
Environmental Research and Public Health

MDPI

Table S1. Questions performed in the first section of the questionnaire, including the respondent characterization and the general knowledge and personal use of antibiotics, for all the respondents. All the results were expressed as the number of responses (% of total responses).

Question		Number (% total)
Age	18-30 years	**174 (39%)**
	31-40 years	**103 (23%)**
	41-50 years	**108 (24%)**
	≥51 years	**64 (14%)**
Gender	Female	**314 (69%)**
	Male	**123 (28%)**
	Without answer	**12 (3%)**
Socio-professional group	Undergraduate students	**164 (36%)**
	PhD students and researchers	**53 (12%)**
	Lecturers	**126 (28%)**
	Technicians and Other occupations	**106 (24%)**
What is your view of bacterial resistance to antibiotics?	Major public health problem	**374 (83%)**
	Maybe worrying but it doesn't matter greatly	**61 (14%)**
	Do not know enough to answer	**12 (3%)**
	Do not know/no opinion	**2 (0%)**
Antibiotic should be administered for:	Bacterial infections	**280 (62%)**
	Viral infections	**30 (7%)**
	Bacterial and viral infections	**25 (6%)**
	Bacterial and fungal infections	**30 (7%)**
	All	**77 (17%)**
	Do not know/no opinion	**7 (2%)**
Prescribed antibiotic should be used:	Until the whole pack/course has been finished	**395 (88%)**
	Until you feel better	**20 (5%)**
	You're not sure	**23 (5%)**
	Do not know/no opinion	**11 (2%)**
Any leftover antibiotic should be:	Returned to the pharmacy	**199 (44%)**
	Thrown in the garbage	**32 (7%)**
	Stored for future use	**47 (11%)**
	Never left unused	**165 (36%)**
	Do not know/Have no opinion	**6 (1%)**
For 3 days you or a family member have had a fever, headache, runny nose, cough and muscle pain. When you see a doctor, do you…?	Consider the doctor competent if s/he prescribes an antibiotic immediately	**42 (9%)**
	Wait for the doctor to test you before prescribing an antibiotic	**192 (43%)**
	Agree to return to the consultation after 2-3 days to be reassessed	**206 (46%)**
	Not know/Have no opinion	**9 (2%)**

Have you ever obtained/taken an antibiotic without it being prescribed by a doctor?	Yes	**111 (25%)**
	No	**336 (75%)**
	Do not know/no opinion	**2 (0%)**
If you answered Yes to Q9, how many times has this happened in the last year? (n=111)	Once	**83 (758%)**
	2 to 5 times	**17 (15%)**
	More than 5 times	**3 (3%)**
	Do not know/Have no opinion	**8 (7%)**
Where do you usually get antibiotics?	Community pharmacies	**247 (55%)**
	Through your doctor	**185 (41%)**
	Online shop / pharmacy	**3 (1%)**
	Other	**7 (2%)**
	Do not know/no opinion	**7 (2%)**
When you are prescribed an antibiotic, does your doctor explain the importance of taking it correctly?	Always	**238 (53%)**
	Sometimes	**174 (39%)**
	Never	**30 (7%)**
	Do not know/no opinion	**7 (2%)**
Can you recognize the name of some antibiotics and their active ingredient?	Yes	**286 (64%)**
	No	**155 (35%)**
	Do not know/no opinion	**8 (2%)**
Have you ever used leftover antibiotics to treat other family members and/or friends?	Never	**388 (86%)**
	1-5 times	**40 (9%)**
	Several times	**14 (3%)**
	Do not know/no opinion	**7 (2%)**
Do you check the pack for instructions on how to use the antibiotic?	Never	**20 (4%)**
	Sometimes	**151 (34%)**
	Always	**270 (60%)**
	Do not know/no opinion	**8 (2%)**
How clear are instructions for antibiotics use?	Totally clear	**207 (46%)**
	Partially clear	**226 (50%)**
	Not clear	**6 (1%)**
	Do not know/no opinion	**10 (2%)**
How many times in the last year have you taken antibiotics?	None	**215 (48%)**
	1-5 times	**222 (49%)**
	More than 5 times	**5 (1%)**
	Do not know/no opinion	**7 (2%)**
Have you ever correctly taken a pack of antibiotics prescribed by the doctor and had to continue treatment with a different antibiotic?	None	**286 (64%)**
	1-5 times	**159 (35%)**
	More than 5 times	**1 (0%)**
	Do not know/no opinion	**3 (1%)**
Do you have pets?	No	290 (65%)
	Yes	159 (35%)

Source: Authors' own compilation, translated from Portuguese.

International Journal of
Environmental Research and Public Health

Table S2. Questions performed in the second section of the questionnaire, including specific knowledge and experience of the use of antibiotics to treat pets, for the pet owners. All the results were expressed as the number of responses and as a percentage of the total number of responses from pet owners (n=159).

Question		Number (% total)
Have you ever used antibiotics on your pet?	Yes	**74 (47%)**
	No	**83 (52%)**
	Do not know/no opinion	**2 (1%)**
Have you ever medicated your pet with non-prescribed antibiotics?	Yes	**13 (8%)**
	No	**143 (90%)**
	Do not know/no opinion	**3 (2%)**
Do you ever stop your pet's antibiotic treatment?	Yes, as soon as I notice improvements	**1 (1%)**
	Yes, when my pet seems to be perfectly back to normal	**10 (6%)**
	Yes	**6 (4%)**
	When indicated by the veterinary doctor	**96 (60%)**
	Do not know/no opinion	**46 (29%)**
Do you change the dosage or antibiotic during treatment?	Yes	**2 (1%)**
	No	**129 (81%)**
	Do not know/no opinion	**28 (18%)**
How regularly over the past year has your veterinarian prescribed antibiotics for your pet?	Once	**70 (44%)**
	1 to 5 times	**15 (9%)**
	Do not know/no opinion	**74 (47%)**
Do you know that the use of antibiotics in animals is allowed only with a prescription from a veterinarian?	Yes	**102 (64%)**
	No	**43 (27%)**
	Do not know/no opinion	**14 (9%)**
Do you know that antibiotic-resistant bacteria can spread from animals to humans and vice versa?	Yes. I'm fully aware of this fact	**66 (41%)**
	I suspected that there might be some connection	**43 (27%)**
	I had no idea	**38 (24%)**
	Do not know/no opinion	**12 (8%)**
Do you usually discuss with your veterinarian your willingness and availability to administer the antibiotic to your pet correctly and on time?	Yes. Always.	**57 (36%)**
	I do not consider this important for the success of the treatment	**19 (12%)**
	Sometimes	**44 (28%)**
	Do not know/no opinion	**39 (25%)**
Does your usual veterinary clinic display information (e.g. posters, flyers) directed to pet owners about the proper use of antibiotics?	Yes	**63 (40%)**
	No	**73 (46%)**
	Do not know/no opinion	**23 (14%)**

Source: Authors' own compilation, translated from Portuguese.
